# Supplementary material for: Acidification and Nutrient Imbalances Drive Fusarium Wilt Severity in Banana (Musa spp.) Grown on Tropical Latosols
Source: J Fungi (Basel). 2025 Aug 22;11(9):611. doi: 10.3390/jof11090611 (PMC12470300; doi:10.3390/jof11090611)
Supplement: Supplementary file 1 [file jof-11-00611-s001.zip › jof-3812828-supplementary.pdf]

Acidification and nutrient imbalances drive *Fusarium* wilt severity in banana (*Musa* spp.) grown on tropical latosols

Tao Jing [a, †], Kai Li [a, †], Lixia Wang [a, †], Mamdouh A. Eissa [a,b], Bingyu Cai [a], Tianyan Yun [a], Yingdui He [a], Ahmed A El Baroudy [c], Zheli Ding [a], Yongzan Wei [a], Yufeng Chen [a], Wei Wang [a], Dengbo Zhou [a,*], Xiaoping Zang [a,*], Jianghui Xie [a,*]

Table S1 Grading standard of 2nd Chinese national soil survey#

Properties	Very high	High	Medium	Low	pH (1:2.5 soil to water)	Soil
Cation exchange capacity (cmol·kg^{-1})	-	>20.0	10-20	<10	>8.5	Alkaline
Soil organic matter (g·kg^{-1})	>40	30-40	20-30	20-30	7.5-8.5	Low alkaline
Total nitrogen (g·kg^{-1})	>2	1.5-2.0	1-1.5	0.75-1.0	6.5-7.5	Neutral
Available phosphorus (mg·kg^{-1})	>40	20-40	20-10	5-10	5.5-6.5	Low acidity
Available potassium (mg·kg^{-1})	>200	150-200	100-150	50-100	4.5-5.5	Acid soil
Exchangeable calcium (mg·kg^{-1})	>1000	700-1000	500-700	300-500		
Exchangeable magnesium (mg·kg^{-1})	>300	200-300	100-200	50-100		
Available boron (mg·kg^{-1})	>2.0	1.0-2.0	0.5-1.0	0.2-0.5		
Available copper (mg·kg^{-1})	>1.8	1.0-1.8	0.2-1.0	0.1-0.2		
Available zinc (mg·kg^{-1})	>3.0	1.0-3.0	0.5-1.0	0.3-0.5		

[#] China Soil Survey Department (1992); Xi and Zhang (1994).

Table S2 The maximum (Max), minimum (Min), and mean values of soil quality indicators for Hainan soils

Grade[#]	Max	Min	Mean	SD	CV
pH	6.79	3.64	4.93	0.69	14
Soil organic matter (g·kg^{-1})	46.06	7.85	23.97	8.14	34
Total nitrogen (g·kg^{-1})	1.75	0.33	1.15	0.38	33
Available phosphorus (mg·kg^{-1})	654	6.7	154	136	89
Available potassium (mg·kg^{-1})	1685	12.8	495	330	67
Exchangeable calcium (mg·kg^{-1})	1369	80	546	312	57
Exchangeable magnesium (mg·kg^{-1})	1774	58	480	424	88
Available copper (mg·kg^{-1})	3.99	0.40	1.72	0.91	52
Available zinc (mg·kg^{-1})	44.26	0.98	6.55	7.43	114
Available boron (mg·kg^{-1})	4.46	0.42	1.34	0.88	65
Cation exchange capacity (cmol·kg^{-1})	15.95	2.81	9.78	2.50	26

SD = Standard deviation, n=47, CV = Coefficient of variation%.

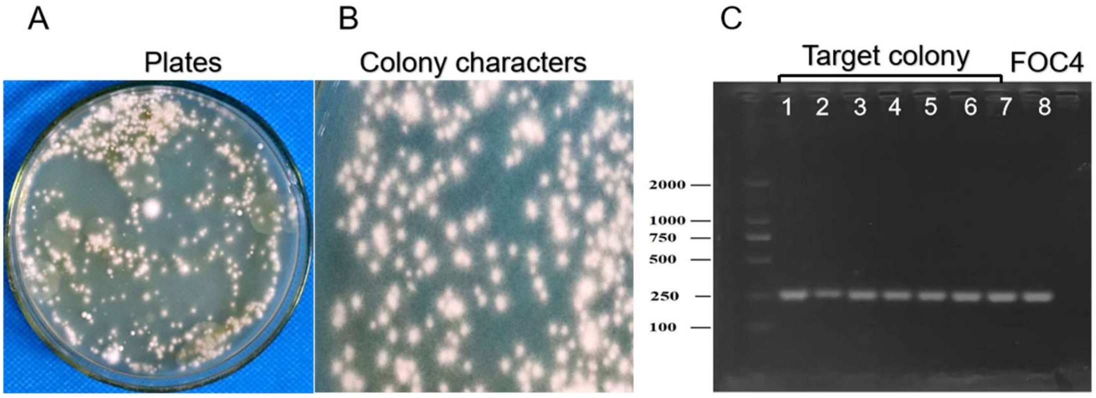

Figure S1: A and B represent the typical colony characteristics on the selective medium, while C is the electrophoresis image of PCR verification.

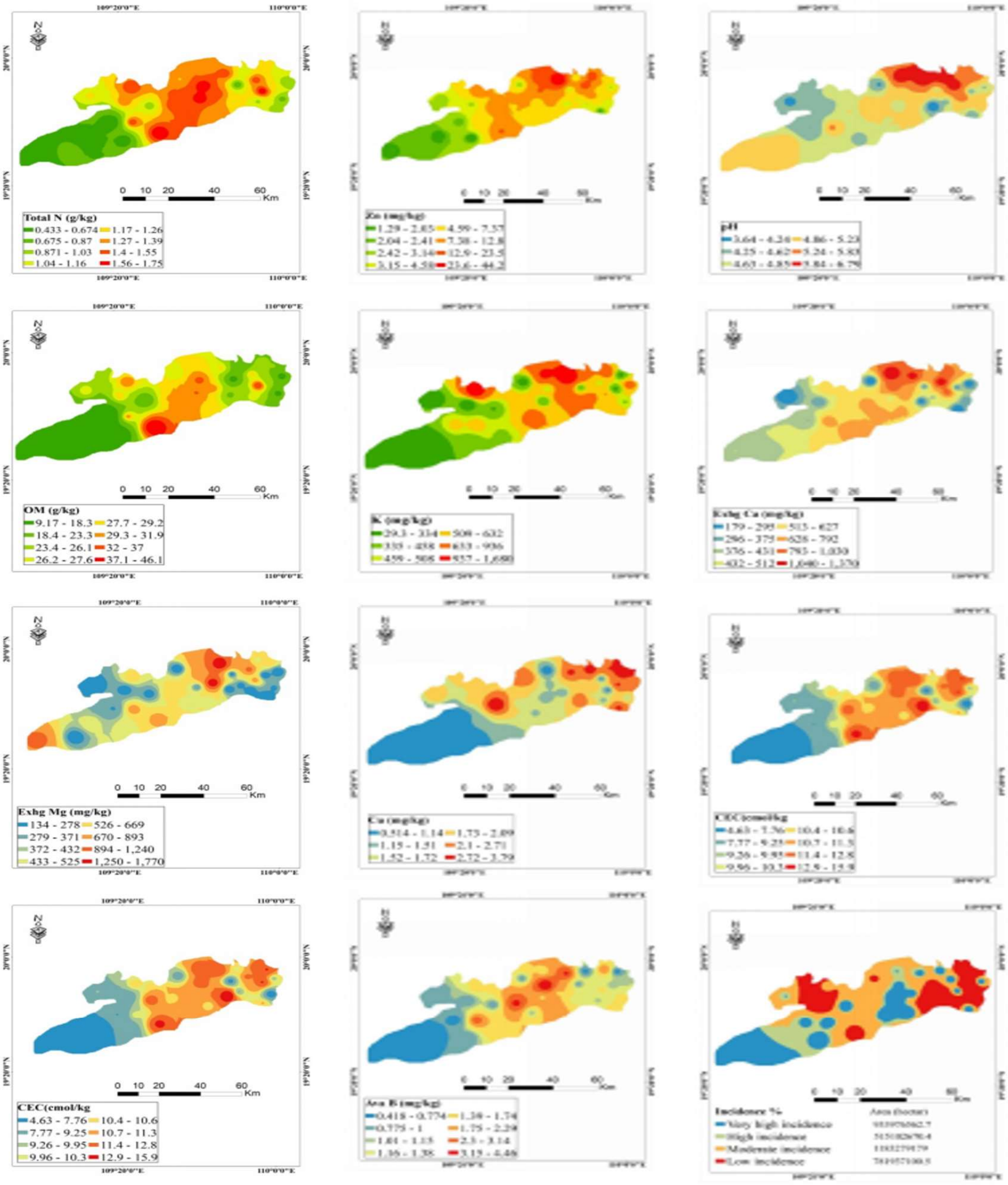

Figure S2. Soil quality classes and Panama disease incidence (PDI) of the study area in Hainan banana orchards (n=47), CEC = Cation exchange capacity. Exgh = exchangeable, and SOM = soil organic matter.
